# Changes in Protein Structural Motifs upon Post-Translational Modification in Kidney Cancer

**DOI:** 10.3390/diagnostics11101836

**Published:** 2021-10-04

**Authors:** Dmitry Tikhonov, Liudmila Kulikova, Vladimir Rudnev, Arthur T. Kopylov, Amir Taldaev, Alexander Stepanov, Kristina Malsagova, Alexander Izotov, Dmitry Enikeev, Natalia Potoldykova, Anna Kaysheva

**Affiliations:** 1 Institute of Mathematical Problems of Biology RAS—The Branch of Keldysh Institute of Applied Mathematics of Russian Academy of Sciences, 142290 Pushchino, Russia; dmitry.tikhonov@gmail.com (D.T.); likulikova@mail.ru (L.K.); 2 Institute of Theoretical and Experimental Biophysics, Russian Academy of Sciences, 142290 Pushchino, Russia; volodyarv@mail.ru; 3 V.N. Orekhovich Institute of Biomedical Chemistry, 119121 Moscow, Russia; a.t.kopylov@gmail.com (A.T.K.); t-amir@bk.ru (A.T.); aleks.a.stepanov@gmail.com (A.S.); izotov.alexander.ibmc@gmail.com (A.I.); kaysheva3@gmail.com (A.K.); 4 Institute of Urology and Reproductive Health, Sechenov University, 119121 Moscow, Russia; enikeev_dv@mail.ru (D.E.); potoldyko-vanv@gmail.com (N.P.)

**Keywords:** kidney cancer, post-translational modifications, active environment, molecular dynamics

## Abstract

Post-translational modification (PTM) leads to conformational changes in protein structure, modulates the biological function of proteins, and, consequently, changes the signature of metabolic transformations and the immune response in the body. Common PTMs are reversible and serve as a mechanism for modulating metabolic trans-formations in cells. It is likely that dysregulation of post-translational cellular signaling leads to abnormal proliferation and oncogenesis. We examined protein PTMs in the blood samples from patients with kidney cancer. Conformational changes in proteins after modification were analyzed. The proteins were analyzed using ultra-high resolution HPLC-MS/MS and structural analysis was performed with the AMBER and GROMACS software packages. Fifteen proteins containing PTMs were identified in blood samples from patients with kidney cancer. For proteins with PDB structures, a comparative analysis of the structural changes accompanying the modifications was performed. Results revealed that PTMs are localized in stable and compact space protein globule motifs that are exposed to a solvent. The phenomenon of modification is accompanied, as a rule, by an increase in the area available for the solvent of the modified amino acid residue and its active environment.

## 1. Introduction

Systems biology enables the integration of data on molecular changes in the body in health and disease. There has been a growing interest in understanding the processes that regulate signaling pathways and, as a consequence, determining the onset of metabolic pathway reprogramming and changes in the cell phenotype during ontogenesis. Changes in cellular phenotype are among the most striking programmed biological events that occur in a healthy cell and include changes in the stages of the cell cycle from prophase to anaphase, de novo gluconeogenesis, differentiation and maturation of a cell into a mature phenotype, and the processes of cells along the path of pathogenesis. Multidimensional omics data on the level of gene expression, transcript content, protein and metabolite composition and content provide a vast array of knowledge that requires further investigation to understand the molecular events accompanying the pathological process [1]. It is important to study dynamic (reversible) events at the molecular level, given that these events likely make a decisive contribution to the uncontrolled inversion of metabolic events during pathogenesis. These dynamic events largely consist of post-translational modifications (PTMs) of proteins [2,3].

The transformation of a healthy cell into a neoplastic cell is accompanied by many signaling events that are regulated by a network of protein modifications [4]. PTM is a phenomenon that modulates the biological activity of a protein and influences the regulation of metabolic transformations in the body. It has been suggested that the pathological process is a time-determined cascade of protein modifications. The cascade of modifications itself, or the signature of post-translationally modified proteins, is specific for each pathophenotype [3].

Recent work has demonstrated the importance of studying the signature of post-translationally modified proteins in oncology [5,6,7,8,9,10] and endocrinology [11,12]. It should be noted that the role of post-translational signaling in reprogramming a healthy phenotype into a pathological one has not yet been elucidated. The biological significance of most types of PTMs in pathogenesis remains unclear. Nevertheless, the most significant protein modifications (phosphorylation, acetylation, methylation, and ubiquitination) have been successfully integrated into the molecular understanding of the pathological process [4].

According to the Global Cancer Observatory (WHO), at least 430,000 cases of kidney cancer were identified globally in 2020. In these cases, morbidity remains similar to other cancers and is more likely due to population density; approximately 36% of patients identified were located in Asia, 32% in Europe, and 18% in North America [13].

In our earlier work, we performed a comparative analysis of the differences in protein content in blood samples from patients with kidney cancer (as well as three other oncopathologies) compared with samples from conditionally healthy study participants (Supplementary Materials) [14]. We found proteins in blood samples from kidney cancer patients significantly different in content from the comparison group (healthy participants), including C4b-binding protein beta chain, galectin-3-binding protein, complement C4-B, phosphatidylinositol-glycan-specific phospholipase D, angiotensinogen, complement C5, C4b-binding protein alpha chain, gelsolin, plasma kallikrein and plasma protease C1 inhibitor. The revealed proteins are participants in the regulation of inflammatory response (GO: 0050727), transport (GO: 0006810) and regulated exocytosis (GO: 0045055).

In this study, we draw the reader’s attention to the presence of PTMs in proteins that are specific to a group of blood samples from patients with kidney cancer. Specific to the pathology post-translational protein modifications were identified, and 15 modified peptides for 15 proteins in kidney cancer were analyzed. For proteins with annotated three-dimensional structures in the Protein Data Bank (PDB) database, a comparative analysis of structural changes via molecular dynamics simulations was performed using the AMBER and GROMACS software packages.

## 2. Materials and Methods

### 2.1. Demography

The study population comprised a group of patients with Stages I–IV of kidney cancer (*n* = 45, aged 56.7 ± 9.8 years) and the control group consisted of 40 healthy volunteers (aged 43.5 ± 14.5 years) with no previous history of oncological disease (Supplementary material Table S1). All groups under consideration were sex-aligned. Patients and healthy volunteers provided written consent to participate in the study, which was approved by the local ethical committee of Sechenov University (Protocol No. 10–19, 17 July 2019) according to the WMA Declaration of Helsinki for ethical principles for medical research involving human subjects.

### 2.2. Sample Preparation for MS Analysis

Blood plasma (40 µL) was mixed with a denaturation solution (0.1% deoxycholic acid sodium salt, 6% acetonitrile, and 75 mM triethylammonium bicarbonate, pH 8.5) and proteins were reduced with 10 mM TCEP (tris-(2-carboxyethyl)phosphine) for 20 min at 40 °C. Further, reduced proteins were alkylated with 0.2% 4-vynylpyridine solution in 30% isopropanol for 30 min at ambient temperature. Prior to digestion, samples were diluted with to 500 µL of 75 mM triethylammonium bicarbonate to adjust pH and to decrease urea and deoxycholic acid concentrations. Digestion was performed with trypsin (200 ng/µL supplemented with 30 mM acetic acid) in two stages: at the first stage, trypsin was added at a ratio of 1:50 (*w*/*w*) and the reaction was incubated for 3 h at 37 °C. In the second stage, the enzyme was added at a ratio of 1:100 (*w*/*w*) and the reaction was incubated at 37 °C for 12 h.

### 2.3. Mass Spectrometry Protein Registration

Mass spectrometry analysis was conducted on a high-resolution Q Exactive-HF mass spectrometer (Thermo Scientific, Waltham, MA, USA) equipped with a nanospray ionization (NSI) source (Thermo Scientific). The selection of mass spectrometry parameters for data acquisition met the requirement of the Human Proteome Organization (HUPO Guidelines, bullet point 9, v 3.0, released 15 October 2019) for the minimal length of the detected peptide for consideration and justification of PE1 proteins (according to the UniProt KB Classification).

Data acquisition was performed in a positive ionization mode in the range of 420–1250 m/z for precursor ions (with resolution R = 60 K) and in a range with the first recorded mass of 110 m/z for fragment ions (with resolution of R = 15 K). Precursor ions were accumulated for a maximum integration time of 15 ms, and fragment ions were accumulated for a maximum integration time of 85 ms. The top 20 precursor ions with a charge state between z = 2+ and z = 4+ were collected and triggered for fragmentation in high-energy collision dissociation mode with a collision energy normalized at 27% and ±20% ramping.

Peptides were separated on an Ultimate 3000 RSLC Nano UPLC system (Thermo Scientific, Waltham, MA, USA) with pre-installed enrichment column (Acclaim Pepmap^®^ (5 × 0.3 mm, 300 Å pore size, 5 µm particle size)) and analytical column (Acclaim Pepmap^®^ analytical column (75 µm × 150 mm, 1.8 µm particle size, 60 Å pore size)). Peptides were loaded 2.5% acetonitrile, 0.1% formic acid, and 0.03% acetic acid at a flow rate of 20 μL/min for 4 min and separated in a linear gradient of mobile Phases A (water with 0.1% formic acid and 0.03% acetic acid) and B (acetonitrile with 0.1% formic acid and 0.03% acetic acid) at a flow rate of 0.3 μL/min using the following elution scheme: started at 2.5% of B for 3 min and was raised to 12% of B for the next 15 min, then to 37% of B for the next 27 min, and to 50% for the next 3 min, then rapid increase to 90% of B for 2 min maintained for 8 min at a flow rate of 0.45 μL/min, and columns system equilibration for the next 13 min in the initial gradient conditions. Mass spectrometric measurements were performed using the equipment of the “Human Proteome” Core Facility (IBMC, Moscow, Russia).

Proteomic data have been deposited to the Mendeley Data V1—Kopylov, Arthur T.; et al. (2021), “post-translational modifications inducing protein motifs structural changes in patients with kidney cancer”, doi: 10.17632/g9k8gg56s7.1.

### 2.4. Protein Identification and Criteria Selection for PTMs

Peak lists obtained after MSConvert (Proteowizard, v3.0.20344) were submitted to the OMSSA (v2.1.9, Proteomics Re-source, Seattle, WA, USA) search engine and processed protein sequence database UniProtKB (taxon—Homo sapiens; 88,703 total number of entries) with a concatenated decoy sequence, generated by the SearchGUI engine (release 3.1.16, Compomics, Gent-Zwijnaarde, Belgium). 

Precursor ions mass tolerance was set to 10.0 ppm window, and fragment ions tolerance was adjusted to 0.01 Da. Trypsin was selected as a specific protease with a maximum of two missed cleavages were allowed with searched peptides. Modifications of acetyl (K), phospho (S), phospho (T), phospho (Y), and Gly-Gly (K) were selected as variable modications. Peptides and proteins were identified using PeptideShaker v1.16.11 (Compomics, Gent-Zwijnaarde, Belgium) and validated at a 1.0% of false discovery rate on PSM, peptides and protein levels.

Results of PTMs search and identification were curated according to the following criteria: (a) at least 98% confidence for peptide identification, (b) at least 80% of pep-tide sequence coverage by fragmentation spectra, and (c) at least 10 units of D-score for PTM probability. Furthermore, the extracted data were manually curated to left spectra with y/b fragment ion pair, properly indicating the location of exact amino acid residue with a PTM signature.

To consider the detectable PTM moieties as relevant to the cancer phenotype and for the structural and molecular dynamic analysis, they should meet the following criteria: (a) the total set of PTM moieties must be detectable and identified in at least 50% of each cancer phenotype; (b) each PTM moiety should be identified in at least 20% of PTM-carrying subjects.

A comparative analysis of the protein content in blood samples of study participants in kidney cancer significantly differed in their abundance compared to those in the control group (*p* < 0.05) (Supplementary Table S2).

### 2.5. Molecular Dynamics Simulations

For the present study, proteins, that contain peptides with a certain type of modification, were selected from the Protein Data Bank (PDB) [15]. A separate sample was created for each such peptide. The number of proteins with target peptides found in the PDB-bank is different for each peptide sequence, and resulting sets of protein structures consist of 1 to 24 proteins.

From the obtained database, all the motifs with peptides under consideration were selected if the number of individual blocks of the protein molecule included motifs, where the first and last helices were in contact (d ≤ 14 Å). The secondary structure of proteins was determined by the DSSP method by Kabsch and Sander [16]. During the MD experiment, we investigated the change of certain geometry characteristics for PTM-containing structures associated with kidney cancer development, i.e., the interplanar d and minimum r distance between the helices, the angles θ and φ between axis of helices, the area S and the perimeter P of the intersection polygon helices projection. The expected value of these quantities was calculated before the start of experiment, and during the experiment these values were recorded in a time-dependent manner. We studied the stability of the molecular system as a whole and of individual blocks of the protein molecule bearing PTMs before and after modification. The definition of important characteristics of structural blocks are described in previously published studies [15,16,17,18,19].

Protein subunits with lengths less than 200 amino acid residues were selected for the MD simulation. The long (100 ns) MD simulation experiments were carried out using the GROMACS package, v2020.4 [20], with a modified GROMOS 54A7 force field [21,22], a 2 fs integration time step, and imposed 3D periodic boundary conditions. A 12 Å spherical cut-off function was used to truncate the van der Waals interactions. Electrostatic effects were treated using the particle-mesh Ewald summation [23] (real space cutoff of 12 Å). The temperature and pressure of the systems were maintained by the V-rescale coupling method [24] at 311 K and the Berendsen coupling method [25] at 1 bar. MD simulations were performed using a simple point-charge (SPC) water model. A certain number of solvent molecules were replaced by Na^+^ or Cl^−^ ions to make each system electrically neutral. Prior to the MD simulation, all systems were subjected to energy minimization (1000 conjugate gradient steps) and subsequently heated to 311 K within 0.2 ns. The protein and solvent molecules were separately coupled. The MD simulation of each system was repeated at least three times by the random assignment of the initial velocities.

The computed MD trajectories were analyzed using the original GROMACS utilities. The dynamic behavior and stability of the studied systems were assessed by root-mean-square deviation and solvent-accessible surface area (SASA) calculation using the GROMACS built-in tools. Preferred conformations of native and post-translationally modified proteins by backbone were determined using the cluster module from the GROMACS package (GROMOS clustering method) [26]. Applied cut-off values 1.3 Å, 5.5 Å, and 3.5 Å were in the clusterization procedure for native and PTM ITAX (PDB ID 1N3Y), RPB1B (PDB ID 5FLM), and DIH7 (PDB ID 6RZA) proteins, respectively, and the largest cluster was extracted. The major conformations were aligned using PyMol software (Schrodinger, LLC, New York, NY, USA).

Second MD experiment: The modeling of the molecular dynamics and the subsequent analysis of the results, were carried out using the Amber11 program package [27]. Modeling was performed without considering the explicit water environment in the force field AMBER ff03 [28] at 300 K. As a result, molecular trajectories of the experimental proteins were calculated. In each case, the energy of the system was minimized at a fixed coordinates the polypeptide chain atoms to order the atomic actions. Then, the molecular system was heated to the selected temperature and molecular dynamic trajectory with a duration of 0.5 ns was generated. The interval between calculations was 0.005 ns. The resulting molecular tracts were visualized using the VMD v1.9.1 software package [29]. Free energy of the complexes was calculated using the generalized Bohrn method [30]. The calculation of the distances between amino acid atoms was performed using the CPPTRAJ package, which is part of the AMBER v11 package. The geometry of secondary structure was characterized in accordance with the generally accepted classification method proposed by Kabsch and Sander. Using the same method, the accessible surface of contact with the solvent was determined.

## 3. Results

### 3.1. Post-Translational Protein Modifications

Mass spectrometric analysis of possible modifications of the identified proteins was carried out for five biologically significant PTMs: phosphorylation of serine, threonine, and tyrosine; acetylation of lysine; N-terminal acetylation; and lysine ubiquitylation. After validating the resulting PTM identifications, modifications associated with oncopathology were not detected in the control group (Table 1).

### 3.2. Structural Analysis of Proteins Carrying PTMs

Supersecondary motifs, consisting of two elements of the secondary structure, and characterizing by the unique folding of the polypeptide chain in space, are in the focus of attention of researchers. Structural motifs can be used as stable structures in studies to predict the tertiary structure of a protein [31]. Earlier [19], we investigated geometric characteristics of double-helical structures. Important characteristics of helical pairs include inter-helical distances, torsion angles between helical axes in helical pairs, the number of amino acids between helices, the length of the helix, and the area and perimeter of intersection of the helical protrusions. Previously [32], based on the geometric and spatial packing characteristics, we formulated rules for the classification of double-helix motifs.

Comparative analysis of peptides stability in studied of proteins compare to those unrelated in origin makes it possible to determine the similarity of supersecondary motifs. For this, we solved the problem of conformational matrix determination by organizing samples of protein structures for each of the 8 PTM-containing peptides. The number of found in the PDB bank proteins, that contains the studied peptide sequences, differed for each sample. The set of peptide structures consisted of 1 to 24 proteins (Table 2, N_prot_ column). The MD experiment revealed that all characteristics of the studied motifs (before and after modification) were within the permissible values over the time of experiment, indicating that the peptide remains intact. It should be noticed that special attention was paid to motifs, where strong interhelical interactions were established. In these instances, the axes of helices intersect and the distance between them is small (the helices are in contact), and values of the area and perimeter of intersection of the projections are not zero. Consequently, we focused on structural motifs (e.g., α-α-corners) that are more stable. Thus, we deduced the L- and V-structures from detailed considerations (the axes of the spirals in these structures do not intersect (d ≠ r, d < r), and the area and perimeter of the intersection of the helices have zero or close to zero values).

First, we performed structural analysis of the amino acid environment, where modifications were found. Upon modification, the change in the total area of active environment accessible to the solvent induced by the modified amino acid was observed (Table 2). For the majority of samples, the modification increases the area of the identified residue and its active environment accessible to the solvent. Only two samples did not follow this trend; instead, the total area of the active environment and the identified residue accessible to the solvent decreased negligibly after modification. These samples, containing ILIVITDGK and LEPIATEVWLINK peptides, consisted of one and five proteins, respectively.

Table 2 summarizes the result of data processing of all the detected and selected PDB protein molecules containing the corresponding target amino acid fragments. In each instance, when the number of proteins containing the target amino acid fragment was ≥2, the average value of the a.a. area available to the solvent is always less than the average value of the modified a.a. area available to the solvent (see Columns VN and VM). Thus, it can be argued that a.a. with PTMs in protein structures sampled here are exposed to the solvent, and the modification always increases the surface area of the amino acid residue available to the solvent. The surface area of the modified amino acid available to the solvent always exceeds that of the unmodified residue, sometimes by more than double (Table 1). The active environment of the modification was determined for each of the selected protein molecule. We define the active environment as an adjacent to the PTM amino acid, which changes the area available for the solvent after modification. Thus, we determined the amino acid context alterations in the area exposed to the solvent, their coordinates in the amino acid sequence, and the number of such amino acids. Having isolated these amino acids, we determined how the total area accessible to the solvent changed (Table 1; Columns UN and UM). The average total area after modification is frequently larger than that if unmodified amino acids are accommodated, but this difference is not as pronounced as the difference in the areas of single amino acid before and after modification. We found several instances, when PTM significantly increased the solvent-accessible area of the modified amino acid, but the total solvent-available area across all amino acid residues was equal to or less than that of unmodified amino acid chains. Thus, it appears that the active environment “tries to level” the increase in the area of the modified amino acid accessible to the solvent.

### 3.3. Molecular Dynamics Simulation of Protein Molecules Containing PTMs Associated with Kidney Cancer

**Figure 1:**
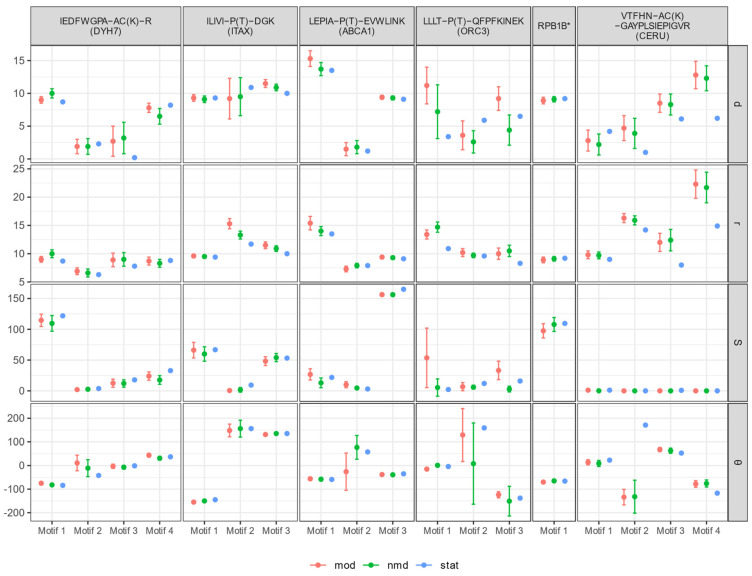
Calculated geometrical features of protein motifs with PTMs. Each motif is characterized by the estimated geometrical features for the intact molecule (stat; blue color), calculated results of MD for the unmodified molecule (nmd; green color), and calculated features for the modified molecule (mod; red color). The right axis indicates (**d**)—inter-planar distance between helices, (**r**)—minimal distance between helices, (**S**)—area of the polygon of the helices projections intersection, and (**θ**)—torsion angle between the axes of the helices. The OX axis shows the motifs of the structures containing the PTMs. Table 3 contains detailed information on the localization (Locus) of the identified structural motives. *VPHPLEH−AC(K)−IIIR (RPB1B).

To study the dynamic behavior of the molecular system as a whole, as well that of individual blocks of a protein molecule bearing PTMs, before and after modification, we performed a molecular dynamics (MD) simulation experiment. Through the MD experiment, we investigated the change in certain characteristics describing the geometry of structures containing PTMs: the interplanar and minimum distance between the helices, the angles θ and φ between the axis of the helices, and the area and perimeter of the polygon of intersection of the projections of the helices. These values were calculated before the start of the experiment and then recorded at certain time intervals during the experiment. We studied the stability of the molecular system as a whole and of individual blocks of protein molecules with PTMs. In this case, the number of individual blocks of the protein molecule included motifs, where the first and the last helices of such block were in contact (d ≤ 14 Å). The calculated geometric characteristics of protein motifs with a PTM fragment are demonstrated in Figure 1. Notably, the closer the values of the studied quantity before and after modification were to the initial value, and the smaller the value of the root-mean-square deviations, the more stable the structure.

It is clear that a helical pair containing PTMs in RPB1B (with the coordinates of the first helix (40–57) and the second helix (83–112)) exhibits stability both before and after modification (Figure 1) and represented as an α-α-corner structure, judging by the initial values: d = r = 9.2 Å, the torsion angle θ = −66°, the area of the polygon of intersection of the projections of the helices S = 109.5 Å^2^, and perimeter P = 42.7 Å. However, based on the empirical values and their standard deviation, the range of values is wider after modification introduction. This indicates that the modified structure was relatively less stable. However, in both cases, the structure does not fall apart; the distances, angles, and other characteristics between the helices remains connections in the structure preserved.

## 4. Discussion

PTMs play an important role in the regulation of protein functions. Covalent binding of a small chemical group to amino acid side chain radical leads to variations in physicochemical properties of proteins, their conformation, ligand-binding ability, and functional activity [33]. PTM-carrying proteins can cover 50–90% of the total proteome in the human body [34]. Several types of PTMs can be found in the same protein, and different types of PTMs have different effects. Revelation of protein PTMs signature at different phenotypic conditions offers a great insight in studying the range of functional regulations underlying complex mechanisms of vital activity. With the development of mass spectrometry in recent years, it has become possible to reliably identify modifications of individual proteins.

Here, we identified PTMs for proteins that were unique for patients with kidney cancer. To date, there are not so many reports on the effect of protein modifications on the development of kidney cancer or oncological diseases in common. However, researchers have associated the functional activity of most intact proteins with oncogenesis.

In the present study, we performed a comparative HPLC-MS/MS analysis of proteins with PTMs specific for patients with kidney cancer. Using molecular dynamics experiment, we attempted to examine the role identified modifications in maintenance of three-dimensional structure stability. Since PTMs often affect the functional activity of protein (in particular, phosphorylation and carboxylation), it is imperative to confirm the preservation of the PTM-bearing protein global stability. Otherwise, dramatic change of protein structure (i.e., molten globule) may trigger the cellular mechanisms of proteins elimination and utilization. For this purpose, we analyzed the following characteristics of protein fragments in unrelated structures: the similarity of blocks, where the studied peptide sequences are located and the effect of the amino acid modification on the accessibility of the local 3D protein region to the solvent and on the stability of the motif and the entire protein structure as a whole.

Actually, MD experiments showed that modification of amino acids did not lead to a complete rupture of bonds and disintegration of motifs or the entire protein molecule. Based on these results, we managed to study three globular structures out of eight proteins with and without PTM in conditions close to native microenvironment (water environment with physiological electrolyte ions). Structures stability and geometry elucidation were performed within 100 ns duration in three times repetition by molecular dynamics simulations in GROMACS package. The limitation of the Gromacs MD simulation package is the lack of compact globular structure of proteins and characteristic fibrillar structures. In addition, it has been showed that some subtle changes in secondary structure elements can be observed for whole protein structures, which are not dramatic for the three-dimensional folding of the protein.

**Figure 2:**
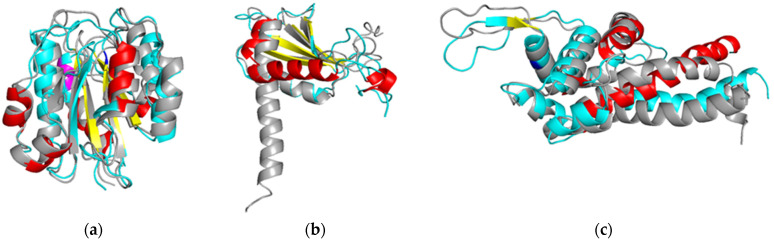
Superpositions of the major conformations of intact and post-translationally modified proteins. Intact and post translationally modified structures are presented as grey and cyan colors, respectively; the site of modification is marked in blue. α-helix, 3_10_-helix, β-strand changed in the post-translationally modified proteins and are marked in red, magenta, and yellow, respectively. (**a**) ITAX, (**b**) RPB1B, (**c**) DYH7.

Particularly, for integrin alpha-X protein (ITAX, PDBID 1N3Y) we observed β-sheet elongation in coordinates (Q40–Q45: Q40–F46), β-sheet relaxation(Q51–F54), displacement and shortening of the α-helix (F56–S62: F55–R61), elongation of the α-helix (P65–A70: P65–S71), displacement and elongation of the β-sheet (A104–V108: K105–T111), and elongation of the α-helix (K176–F187: F172–F187). Changes in the secondary structure in the MD experiment induce allosteric modulation of the alpha X integrin receptor (Figure 2a). Interestingly, this protein is reported likely to be associated with the development of oncological pathology. Integrin alpha-X belongs to the integrin family and is an extracellular matrix receptor. In recent years, it has been shown that overexpression of ITAX umbilical vein endothelial cells is likely involved in angiogenesis of dendritic cells and increases the rate of proliferation, migration, angiogenesis, and tumor growth [35]. The ITAX protein activates the PI3k/Akt-mediated signaling pathway, which leads to an increased expression of c-Myc, vascular endothelial growth factor-A (VEGF-A), and VEGF receptor 2 (VEGFR2). During the kidney cancer development, increased expression of ITAX is associated with a negative survival prognosis [36]. The observed structural changes in the elements of the secondary structure may modulate the biological function of the protein. On the other hand, ITAX has a low cancer specificity since the expression level is close to the basal level in most of cancer phenotypes. However, closer examination showed, that several ITAX polymorphism may significantly enhance the overall risk of IgA nephropathy [37] that may contribute in renal cell carcinoma and mesothelioma especially in elderly patients [38].

Close examination of DNA-directed RNA polymerase II Subunit RPB11—a protein (RPB1B, PDBID 5FLM), revealed β-sheet shortening (coordinates I19–K23: I21–K23), β-sheet elongation (C31–N36: A30–N36, L57–Y61: L57–K62), as well as shortening in the axis of the α-helix and (P83–D111: P83–F105; Figure 2b). It was known, that this protein is implicated in RNA processing, chromatin remodeling, exosome processing, and RNA pol II transcription elongation/pausing [39]. DNA-dependent RNA polymerase catalyzes the transcription of DNA into RNA. Rpb3/Rpb11 proteins form a heterodimeric complex that is involved in promoter recognition and is involved in the regulation of transcription of almost all pre-mRNAs in eukaryotic cells [40]. However, there is no convincing evidence of changes in the level of expression or translation of Rpb11 in cancer in the literature.

The last attractive protein for which the MD experiment (GROMACS package) was performed under conditions close to native is dynein heavy Chain 7 (DYH7), which constitutes the axoneme dynein heavy chain and involved in cell motility. Genomic variations in members of the DYH family have been reported for various types of malignant tumors [41]. The study of Zhu et al. (Department of Gastrointestinal Surgery, Shanghai, China) analyzed clinical and genomic data from 132 patients with gastric cancer and found association between somatic mutations in DYH gene family and the effectiveness of response to chemotherapy. Compared to patients with a wild-type of DYH genes (*n* = 59), a high proportion of patients with mutations in the DYH genes (*n* = 73) (55.9% vs. 80.8%) responded to chemotherapy (*p* = 0.002) and DYH mutations were correlated with better survival (*p* = 0.027) [41]. Although there is no string evidence about association of DYH7 with the exact cancer phenotype, its main contribution can be related to the transport of misfolded proteins and assembling of aggresomal complexes abundantly produced in the advanced cancer due to expanded inflammatory and chemotherapy [42].

We observed, that DYH protein structure is characterized by a shortening of alpha helix (M88–E96: M88–Q92) and elongation of α-helix (P104–Y114: P104–I115). The occupancy of conformations for a given protein during the simulation is of interest. The intact structure exists in three major conformations with similar populations (in time scale). The modified form of dynein has only one stable conformation, which limits the flexibility of modified structure compared to the intact structure (Figure 2c).

It is also important to note the important biological role of other proteins considered in this study. The ATP-binding cassette Sub-Family A member 1 (ABCA1) uses the energy obtained from ATP hydrolysis to transport substrates through the membrane against their concentration gradient [43]. The role of the ABCA1 transporter in cancer development is related to its ability to export drugs or toxins and transport lipids such as cholesterol, which may affect the proliferation, survival, and migration of cancer cells [44,45,46,47].

Arf-GAP with coiled-coil (ACAP1) belongs to the Arf GAP family and is involved in GTP hydrolysis [48]. Proteins of this family are triggered in oncogenes and control cell proliferation and migration [49,50,51]. The latter effects are thought to be mediated by the coordination of changes in actin remodeling and membrane movement [52].

Abnormal spindle-like microcephaly associated protein (ASPM) is required for mitotic spindle function during cell division. This protein is clinically significant for predicting oncopathology onset and early development. The clinical and prognostic value of ASPM has been demonstrated by the Zhenglin Xu et al. (Department of Urology, Zhejiang, China) for urologic cancers, including prostate cancer (PC) and renal cell carcinoma (RCC) [53]. ASPM gene expression is associated with Gleason score, Stage T, and Stage N PCa (*p* < 0.05), and has also been significantly associated with old age and late stage TNM in RCC [53].

It is also worth noting that other proteins for which modifications have been identified can participate in oncogenesis, based on panoply of reported investigation. Apolipoprotein A-I (apoA-I) is the main component of high-density lipoproteins (HDL) and is a multifunctional protein involved in the transmission of cholesterol and regulation of inflammatory and the immune response. Many studies suggest that the blood level of apoA-I protein changes during the development and progression of various types of cancer [54,55,56]. Serum apoA-I level is thought to be promising biomarker for cancer risk assessment, early diagnosis, and cancer prognosis stratification [55,56,57]. It should be noticed, that apoA-I is yet unfavorable marker of renal cancer, but some polymorphisms credibly increase the risk of renal carcinoma and enhance distal malignancy, which might be of special interest due to such polymorphisms, assumingly, may cause non-specific PTMs localization [58].

Ceruloplasmin is a cofactor for various physiological enzymatic reactions and involved in copper transportation [59]. High levels of ceruloplasmin expression have been defined in various cancers, such as thyroid carcinoma [60], melanoma [61], and kidney cancer [62]. Transcription factors (TFs) are the driving force behind cell proliferation in cancer cell lines; however, the contribution of specific TFs to oncogenesis and the mechanism TF gene regulation remain unclear. Galli et al. considered the PAX8 TF as a candidate oncogene in renal cell carcinoma (RCC) and demonstrated that this TF activates the expression of metabolic genes, including ceruloplasmin, by binding distal enhancer elements and recruiting histone acetylation activity [63].

Protein A-kinase anchor protein 9 (AKAP9) has not been annotated yet, despite it plays an important role in the cellular signaling regulation. AKAP family proteins mediate signal transduction through cAMP-dependent protein kinases [64,65]. Changes in the AKAP9 protein expression and its mutations have been associated with the development of systemic diseases, including chronic heart failure (amino acid substitution Ser1570Lys) [66], various types of cancer [67,68,69], and immune system disorders [70]. Unlike the association with colorectal cancer, there is almost no information about implication of AKAP9 in renal cancer, however, data of observational study presented in the Human Protein Atlas indicate that high expression of AKAP9 (median FPKM = 3.97 for survived and median FPKM = 3.27 for dead patients) is favorable in patients with renal carcinoma [71].

There was a significant increase in the expression of AKAP9 in tumor tissues compared to the control tissue samples. Indeed, AKAP9 level is correlated with the depth of tumor infiltration and metastasis. High expression of AKAP9 is also associated with low survival in patients with colorectal cancer (CRC) [67]. In cultured CRC cells, knockdown of AKAP9 gene follows to inhibition of cell proliferation, invasion, and migration, whereas in vivo AKAP9 deficiency leads to suppression of CRC tumors and metastasis [67].

## 5. Conclusions

The treatment of oncological diseases is difficult due to the lack of reliable approaches to diagnosing the disease, shortcomings in predicting the effectiveness of therapeutic measures [72]. In this regard, the search for candidate non-invasive markers of oncological diseases is extremely promising. To date, many studies are devoted to the study of not only differentially expressed proteins in oncology, but also the identification of disease-specific protein isoforms formed as a result of post-translational protein modification, alternative splicing of protein-coding genes, and genetic polymorphisms leading to amino acid substitutions during translation.

Among more than 300 known post-translational modifications, glycosylation and phosphorylation are most often mentioned in studies in the context of oncogenesis [3]. Glycoproteins are widely reported in the literature as candidate biomarkers of inflammatory and oncological diseases [73]. Secretory forms and membrane-bound glycoproteins are promising targets for non-invasive detection. A new generation of targeted quantitative assays are expected to improve the use of glycoproteins for early disease detection, molecular classification of diseases, and monitoring of therapeutic interventions [73].

In this study, we reported PTMs found at non-specific localizations on molecular surfaces that have not yet been annotated and not detected in normal plasma, thus, suggestively, such PTMs might be atypical. It is assumed that such PTMs can characterize cancer phenotype and the progress of tumorigenesis. However, the majority of such unpacifically modified protein maintain stability as has been shown by MD experiments (Amber and Gromacs).

We assume the new approach for analyzing conformational changes and structural reordering caused by PTM mounting. The analysis takes off a small part of the protein molecule (motif) and the active environment organized by adjacent amino acid residues, and includes most affected by the PTM secondary structural elements. We determined the main structural features of the studied motifs, including spatial coordinates, distances between helices, torsion angle, length, area, and polygonal perimeter of helices within the accounted motifs. It was demonstrated that the modifying moiety was always present outside the protein globule in the solvent environment. Following the MD simulation analysis, we calculated the protein distribution depending on the accommodated PTM type, the end-up solvent-accessible area before and after introducing of PTM, and the active environment that also mutates exhibition to the surrounding solvent. It has been established that the solvent-accessible area of the modified amino acid residue always exceeds that of the intact residue. However, the total accessible area of the active environment, combined with the modified residue, can be equal or marginally smaller than that of the incorporated intact residue. MD simulation revealed that the accommodated PTM moiety did not dramatically change the molecular stability and did not approximate the molten globule state. These results were confirmed for three proteins in the molecular dynamics experiment, when we observe weak changes in characteristics of secondary structure elements at the level of “breathing of the molecule”. Despite the high structural stability, the discovered molecular conformations can contribute significantly to protein function. The proposed approach for analysis of a small spatially stable protein motif and its active environment improves the performance of MD simulation, expands the range of spatial structures of proteins for the analysis, and provides an excellent opportunity to evaluate the influence of PTM-induced functional flexibility on the onset and growth of tumors and improve the relevance of currently underevaluated PTMs.

Employment and inclusion of reliable PTMs accommodated at non-specific sites may improve and empower clinical diagnosis with no obligation of invasive medical tests (for example, biopsy). Certainly, there is a risk of false positive results caused by reasons other than developing (or latent) tumorigenesis since PTMs exchange is tightly related and allied with the comprehensive network of metabolomic reactions. Therefore, obviously, searching of such PTM forms is not the only insight and decision, and requires deeper consideration of lifestyle, dietary preferences, physical activity, and life conditions. Due attention to these important aspects may shorten the list of the observable “bizarre” PTMs leaving only those specifically recognized either for general processes of tumorigenesis onset or, perfectly, for certain type of oncophenotypes. Hence, our studies are the pilot stage challenging the discovery of non-canonically localized PTMs and their impact on structural stability of carrying proteins, that would require further deeper insights and percolations to keep a small fraction credibly specific for oncogenesis.

## Figures and Tables

**Table 1:** Results of mass spectrometric identification of PTM in proteins present in blood samples from patients with kidney cancer.

Sequence with PTM	Gene Name	Protein Name	Frequency, %
SPAGPAATPAQAQAAS-P(T)-PRK	TCOF	Treacle protein	56
QE-AC(K)-EQVSLR	AKAP9	A-kinase anchor protein 9	49
ILIVI-P(T)-DGK	ITAX	Integrin alpha-X	37
LEPIA-P(T)-EVWLINK	ABCA1	ATP-binding cassette sub-Family A, Member 1	35
LSAQA-P(S)-LKR	JKIP1	Janus kinase and microtubule-interacting protein 1	35
Y-AC(K)-DPVTVVVDDLR	ACAP1	Arf-GAP with coiled-coil	33
LYLAVKNAN-AC(K)	ASPM	Abnormal spindle-like microcephaly-associated protein	30
IEDFWGPA-AC(K)-R	DYH7	Dynein heavy chain 7	23
LILEHQE-AC(K)	SCLT1	Sodium channel and clathrin linker 1	12
IK-P(Y)-APISGGDHAEVDVPK	TENA	Tenascin	7
P(Y)-HWEHTGLTLR	APOB	Apolipoprotein B-100	5
VPHPLEH-AC(K)-IIIR	RPB1B	DNA-directed RNA polymerase II, Subunit RPB11-a	5
AC(K)-WQEEMELYR	APOA1	Apolipoprotein A-I	2
VTFHN-AC(K)-GAYPLSIEPIGVR	CERU	Ceruloplasmin	2
LLLT-P(T)-QFPFKINEK	ORC3	Origin recognition complex Subunit 3	2

**Table 2:** Change in the average value of the amino acid (a.a.) area accessible to the solvent and its immediate environment for each target amino acid fragment before and after modification (Å2).

Gene Name	N_prot_	VN	VM	UN	UM	LOC	PTM SEQQ	PROTEIN
ITAX	5	3.92	4.12	49.82	48.54	T6	ILIVITDGK	Integrin alpha-X
ABCA1	1	47.5	49.5	409.7	408.6	T6	LEPIATEVWLINK	ATP-binding cassette Sub-Family A, Member 1
ACAP1	10	170.09	222.4	352.68	321.39	K2	YKDPVTVVVDDLR	Arf-GAP with coiled-coil, ANK repeat and PH domain-containing protein 1
DYH7	1	88.7	123.4	304.8	321.6	K9	IEDFWGPAKR	Dynein heavy chain 7, axonemal
RPB1B	13	76.215	121.877	269.138	297.762	K8	VPHPLEHKIIIR	DNA-directed RNA polymerase II subunit RPB11-a
APOA1	24	87.683	131.825	299.1625	326.867	K1	KWQEEMELYR	Apolipoprotein A-I
CERU	4	91.95	110.725	246.475	247.825	K6	VTFHNKGAYPLSIEPIGVR	Ceruloplasmin
ORC3	5	41.34	88.46	390.64	396,9	T5	LLLTTQFPFKINEK	Origin recognition complex, Subunit 3

NAMTBL—PDB ID; N_prot_: the number of proteins found in the PDB-bank containing the corresponding target amino acid fragment; VN is the average value of the a.a. area available for the solvent (in AMBER, where there is one chain) before modification, Å2; VM is the average value of the area of the modified a.a. accessible to the solvent (in AMBER, where there is one chain), Å2; UN is the average value of the total area of the isolated amino acids of the unmodified chain accessible to the solvent, Å2; UM is the average value of the total area of the isolated amino acids of the modified chain accessible to the solvent, Å2; PTM SEQQ, protein fragment with identified PTM (amino acid sequence); LOC—type of PTM.

**Table 3:** Information on the localization (Locus) of the identified structural motives, Helix A (H_A_) and Helix B (H_B_), the number of alpha-helices (N_helix_) that make up the motive.

Gene Name	PTM SEQQ	Motif 1		Motif 2		Motif 3		Motif 4	
		Locus	N_helix_	Locus	N_helix_	Locus	N_helix_	Locus	N_helix_
DYH7	IEDFWGPA-AC(K)-R	44–110 H_A_ (44–57) H_B_ (104–110)	4	44–128 H_A_ (44–57) H_B_ (122–128)	5	44–86 H_A_ (44–57) H_B_ (77–86)	2	77–96 H_A_ (44–57) H_B_ (89–96)	2
ITAX	ILIVI-P(T)-DGK	81–132 H_A_ (305–316) H_B_ (452–465)	2	81–157 H_A_ (305–316) H_B_ (523–527)	3	12–188 H_A_ (81–90) H_B_ (179–188)	6	–	–
ABCA1	LEPIA-P(T)-EVWLINK	305–465 H_A_ (81–90) H_B_ (125–132)	8	305–527 H_A_ (81–90) H_B_ (125–132)	9	326–545 H_A_ (326–341) H_B_ (529–545)	9	–	–
ORC3	LLLT-P(T)-QFPFKINEK	205–241 H_A_ (205–216) H_B_ (228–241)	2	205–261 H_A_ (205–216) H_B_ (245–261)	3	205–368 H_A_ (205–216) H_B_ (359–368)	9	–	–
RPB1B	VPHPLEH-AC(K)-IIIR	40–112 H_A_ (40–57) H_B_ (83–112)	2	–	–	–	–	–	–
CERU	VTFHN-AC(K)-GAYPLSIEPIGVR	322–516 H_A_ (322–327) H_B_ (510–516)	3	377–565 H_A_ (377–382) H_B_ (557–565)	3	377–672 H_A_ (377–382) H_B_ (667–672)	5	377–856 H_A_ (377–382) H_B_ (851–856)	7

## Data Availability

Correspondence and requests for materials should be addressed to A.K.

## Supplementary Materials

The following are available online at https://www.mdpi.com/article/10.3390/diagnostics11101836/s1, Table S1 Demography; Table S2 List of proteins associated with the development of the kidney cancer.

## References

[1] Subramanian I., Verma S., Kumar S., Jere A., Anamika K. (2020). Multi-Omics Data Integration, Interpretation, and Its Application. Bioinform. Biol. Insights.

[2] Krug K., Mertins P., Zhang B., Hornbeck P., Raju R., Ahmad R., Szucs M., Mundt F., Forestier D., Jane-Valbuena J. (2019). A Curated Resource for Phosphosite-Specific Signature Analysis. Mol. Cell. Proteom..

[3] Mnatsakanyan R., Shema G., Basik M., Batist G., Borchers C.H., Sickmann A., Zahedi R.P. (2018). Detecting Post-Translational Modification Signatures as Potential Biomarkers in Clinical Mass Spectrometry. Expert Rev. Proteom..

[4] Sharma B.S., Prabhakaran V., Desai A.P., Bajpai J., Verma R.J., Swain P.K. (2019). Post-Translational Modifications (PTMs), from a Cancer Perspective: An Overview. Oncogen.

[5] Díaz-Fernández A., Miranda-Castro R., de-los-Santos-Álvarez N., Lobo-Castañón M.J. (2018). Post-Translational Modifications in Tumor Biomarkers: The next Challenge for Aptamers?. Anal. Bioanal. Chem..

[6] Heo K.-S. (2019). Regulation of Post-Translational Modification in Breast Cancer Treatment. BMB Rep..

[7] Zou X., Blank M. (2017). Targeting P38 MAP Kinase Signaling in Cancer through Post-Translational Modifications. Cancer Lett..

[8] Celano M., Mio C., Sponziello M., Verrienti A., Bulotta S., Durante C., Damante G., Russo D. (2018). Targeting Post-Translational Histone Modifications for the Treatment of Non-Medullary Thyroid Cancer. Mol. Cell. Endocrinol..

[9] Shi A.-M., Tao Z.-Q., Li R., Wang Y.-Q., Wang X., Zhao J. (2016). Vimentin and Post-Translational Modifications in Cell Motility during Cancer—A Review. Eur. Rev. Med. Pharmacol. Sci..

[10] Perri A.M., Agosti V., Olivo E., Concolino A., Angelis M.D., Tammè L., Fiumara C.V., Cuda G., Scumaci D. (2019). Histone Proteomics Reveals Novel Post-Translational Modifications in Breast Cancer. Aging.

[11] Cooper A., Woulfe D., Kilic F. (2019). Post-Translational Modifications of Serotonin Transporter. Pharmacol. Res..

[12] Wende A.R. (2016). Post-translational Modifications of the Cardiac Proteome in Diabetes and Heart Failure. Proteom.—Clin. Appl..

[13] International Agency for Research on Cancer. https://gco.iarc.fr/today/home.

[14] Kopylov A.T., Petrovsky D.V., Stepanov A.A., Rudnev V.R., Malsagova K.A., Butkova T.V., Zakharova N.V., Kostyuk G.P., Kulikova L.I., Enikeev D.V. (2021). Convolutional Neural Network in Proteomics and Metabolomics for Determination of Comorbidity between Cancer and Schizophrenia. J. Biomed. Inform..

[15] Berman H.M. (2000). The Protein Data Bank. Nucleic Acids Res..

[16] Kabsch W., Sander C. (1983). Dictionary of Protein Secondary Structure: Pattern Recognition of Hydrogen-Bonded and Geometrical Features. Biopolymers.

[17] Tikhonov D.A., Kulikova L.I., Efimov A.V. (2019). The Study of Interhelical Angles in the Structural Motifs Formed by Two Helices. Math. Biol. Bioinform..

[18] Tikhonov D.A., Kulikova L.I., Efimov A.V. (2018). Analysis of the Torsion Angles between Helical Axes in Pairs of Helices in Protein Molecules. Math. Biol. Bioinform..

[19] Tikhonov D.A., Kulikova L.I., Efimov A.V. (2019). Statistical Analysis of the Internal Distances of Helical Pairs in Protein Molecules. Math. Biol. Bioinform..

[20] Abraham M.J., Murtola T., Schulz R., Páll S., Smith J.C., Hess B., Lindahl E. (2015). GROMACS: High Performance Molecular Simulations through Multi-Level Parallelism from Laptops to Supercomputers. SoftwareX.

[21] Petrov D., Margreitter C., Grandits M., Oostenbrink C., Zagrovic B. (2013). A Systematic Framework for Molecular Dynamics Simulations of Protein Post-Translational Modifications. PLoS Comput. Biol..

[22] Margreitter C., Petrov D., Zagrovic B. (2013). Vienna-PTM Web Server: A Toolkit for MD Simulations of Protein Post-Translational Modifications. Nucleic Acids Res..

[23] Essmann U., Perera L., Berkowitz M.L., Darden T., Lee H., Pedersen L.G. (1995). A Smooth Particle Mesh Ewald Method. J. Chem. Phys..

[24] Bussi G., Donadio D., Parrinello M. (2007). Canonical Sampling through Velocity Rescaling. J. Chem. Phys..

[25] Berendsen H.J.C., Postma J.P.M., van Gunsteren W.F., DiNola A., Haak J.R. (1984). Molecular Dynamics with Coupling to an External Bath. J. Chem. Phys..

[26] Daura X., Gademann K., Jaun B., Seebach D., Van Gunsteren W., Mark A.E., Peggion E. (1999). Peptide Folding: When Simulation Meets Experiment. Angew. Chem. Int. Ed..

[27] Case D.A., Cheatham T.E., Darden T., Gohlke H., Luo R., Merz K.M., Onufriev A., Simmerling C., Wang B., Woods R.J. (2005). The Amber Biomolecular Simulation Programs. J. Comput. Chem..

[28] Duan Y., Wu C., Chowdhury S., Lee M.C., Xiong G., Zhang W., Yang R., Cieplak P., Luo R., Lee T. (2003). A Point-Charge Force Field for Molecular Mechanics Simulations of Proteins Based on Condensed-Phase Quantum Mechanical Calculations. J. Comput. Chem..

[29] Humphrey W., Dalke A., Schulten K. (1996). VMD: Visual Molecular Dynamics. J. Mol. Graph..

[30] Onufriev A., Bashford D., Case D.A. (2000). Modification of the Generalized Born Model Suitable for Macromolecules. J. Phys. Chem. B.

[31] Rudnev V.R., Kulikova L.I., Kaysheva A.L., Efimov A.V., Tikhonov D.A. (2021). Use of the Molecular Dynamics Method to Investigate the Stability of α-α-Corner Structural Motifs in Proteins. Symmetry.

[32] Tikhonov D., Kulikova L., Kopylov A., Malsagova K., Stepanov A., Rudnev V., Kaysheva A. (2020). Super Secondary Structures of Proteins with Post-Translational Modifications in Colon Cancer. Molecules.

[33] Zhang H., Han W. (2020). Protein Post-Translational Modifications in Head and Neck Cancer. Front. Oncol..

[34] Doyle H.A., Mamula M.J. (2001). Post-Translational Protein Modifications in Antigen Recognition and Autoimmunity. Trends Immunol..

[35] Wang J., Yang L., Liang F., Chen Y., Yang G. (2019). Integrin Alpha x Stimulates Cancer Angiogenesis through PI3K/Akt Signaling–Mediated VEGFR2/VEGF-A Overexpression in Blood Vessel Endothelial Cells. J. Cell. Biochem..

[36] Sui Y., Lu K., Fu L. (2021). Prediction and Analysis of Novel Key Genes ITGAX, LAPTM5, SERPINE1 in Clear Cell Renal Cell Carcinoma through Bioinformatics Analysis. PeerJ.

[37] Shi D., Zhong Z., Xu R., Li B., Li J., Habib U., Peng Y., Mao H., Li Z., Huang F. (2019). Association of ITGAX and ITGAM Gene Polymorphisms with Susceptibility to IgA Nephropathy. J. Hum. Genet..

[38] Mimura I., Tojo A., Kinugasa S., Uozaki H., Fujita T. (2009). Renal Cell Carcinoma in Association with IgA Nephropathy in the Elderly. Am. J. Med. Sci..

[39] Willmann K.L., Milosevic S., Pauklin S., Schmitz K.-M., Rangam G., Simon M.T., Maslen S., Skehel M., Robert I., Heyer V. (2012). A Role for the RNA Pol II-Associated PAF Complex in AID-Induced Immune Diversification. J. Exp. Med..

[40] Fang Z.-P., Jiang B.-G., Zhang F.-B., Wang A.-D., Ji Y.-M., Xu Y.-F., Li J.-C., Zhou W.-P., Zhou W.-J., Han H.-X. (2014). Rpb3 Promotes Hepatocellular Carcinoma through Its N-Terminus. Oncotarget.

[41] Zhu C., Yang Q., Xu J., Zhao W., Zhang Z., Xu D., Zhang Y., Zhao E., Zhao G. (2019). Somatic Mutation of DNAH Genes Implicated Higher Chemotherapy Response Rate in Gastric Adenocarcinoma Patients. J. Transl. Med..

[42] García-Mata R., Bebök Z., Sorscher E.J., Sztul E.S. (1999). Characterization and Dynamics of Aggresome Formation by a Cytosolic GFP-Chimera. J. Cell Biol..

[43] Ambudkar S.V. (1998). Drug-Stimulatable ATPase Activity in Crude Membranes of Human MDR1-Transfected Mammalian Cells. Methods Enzymol..

[44] Domenichini A., Adamska A., Falasca M. (2019). ABC Transporters as Cancer Drivers: Potential Functions in Cancer Development. Biochim. Biophys. Acta BBA—Gen. Subj..

[45] Begicevic R.-R., Falasca M. (2017). ABC Transporters in Cancer Stem Cells: Beyond Chemoresistance. Int. J. Mol. Sci..

[46] Aye I.L.M.H., Singh A.T., Keelan J.A. (2009). Transport of Lipids by ABC Proteins: Interactions and Implications for Cellular Toxicity, Viability and Function. Chem. Biol. Interact..

[47] Pasello M., Giudice A.M., Scotlandi K. (2020). The ABC Subfamily A Transporters: Multifaceted Players with Incipient Potentialities in Cancer. Semin. Cancer Biol..

[48] Xia C., Ma W., Stafford L.J., Liu C., Gong L., Martin J.F., Liu M. (2003). GGAPs, a New Family of Bifunctional GTP-Binding and GTPase-Activating Proteins. Mol. Cell. Biol..

[49] Ahn J.-Y., Ye K. (2005). PIKE GTPase Signaling and Function. Int. J. Biol. Sci..

[50] Ye K., Snyder S.H. (2004). PIKE GTPase: A Novel Mediator of Phosphoinositide Signaling. J. Cell Sci..

[51] Ha V.L., Luo R., Nie Z., Randazzo P.A. (2008). Contribution of AZAP-Type Arf GAPs to Cancer Cell Migration and Invasion. Adv. Cancer Res..

[52] Ye K., Hurt K.J., Wu F.Y., Fang M., Luo H.R., Hong J.J., Blackshaw S., Ferris C.D., Snyder S.H. (2000). PIKE: A Nuclear Gtpase That Enhances PI3kinase Activity and Is Regulated by Protein 4.1N. Cell.

[53] Xu Z., Zhang Q., Luh F., Jin B., Liu X. (2019). Overexpression of the ASPM Gene Is Associated with Aggressiveness and Poor Outcome in Bladder Cancer. Oncol. Lett..

[54] Levine A.J., Puzio-Kuter A.M. (2010). The Control of the Metabolic Switch in Cancers by Oncogenes and Tumor Suppressor Genes. Science.

[55] Georgila K., Vyrla D. (2019). Apolipoprotein A-I (ApoA-I), Immunity, Inflammation and Cancer. Cancers.

[56] Jiang Y., Sun A., Zhao Y., Ying W., Sun H., Yang X., Xing B., Sun W., Ren L., Chinese Human Proteome Project (CNHPP) Consortium (2019). Proteomics Identifies New Therapeutic Targets of Early-Stage Hepatocellular Carcinoma. Nature.

[57] Guo S., He X., Chen Q., Yang G., Yao K., Dong P., Ye Y., Chen D., Zhang Z., Qin Z. (2016). The Effect of Preoperative Apolipoprotein A-I on the Prognosis of Surgical Renal Cell Carcinoma: A Retrospective Large Sample Study. Medicine.

[58] Liu Z., Xiao Y., Tang L., Jiang L., Wang Y., Zhang R., Wei Q., Lu Y. (2015). Apolipoprotein A1 −75 G/A and +83 C/T Polymorphisms and Renal Cancer Risk. Lipids Health Dis..

[59] Luza S.C., Speisky H.C. (1996). Liver Copper Storage and Transport during Development: Implications for Cytotoxicity. Am. J. Clin. Nutr..

[60] Kondi-Pafiti A., Smyrniotis V., Frangou M., Papayanopoulou A., Englezou M., Deligeorgi H. (2000). Immunohistochemical Study of Ceruloplasmin, Lactoferrin and Secretory Component Expression in Neoplastic and Non-Neoplastic Thyroid Gland Diseases. Acta Oncol..

[61] Cox C., Teknos T.N., Barrios M., Brewer G.J., Dick R.D., Merajver S.D. (2001). The Role of Copper Suppression as an Antiangiogenic Strategy in Head and Neck Squamous Cell Carcinoma. Laryngoscope.

[62] Allison S.J. (2019). PAX8: A Candidate Oncogene in RCC. Nat. Rev. Nephrol..

[63] Bleu M., Gaulis S., Lopes R., Sprouffske K., Apfel V., Holwerda S., Pregnolato M., Yildiz U., Cordo’ V., Dost A.F.M. (2019). PAX8 Activates Metabolic Genes via Enhancer Elements in Renal Cell Carcinoma. Nat. Commun..

[64] Pidoux G., Taskén K. (2010). Specificity and Spatial Dynamics of Protein Kinase A Signaling Organized by A-Kinase-Anchoring Proteins. J. Mol. Endocrinol..

[65] Dodge-Kafka K.L., Bauman A., Kapiloff M.S. (2008). A-Kinase Anchoring Proteins as the Basis for CAMP Signaling. Handb. Exp. Pharmacol..

[66] Chen L., Marquardt M.L., Tester D.J., Sampson K.J., Ackerman M.J., Kass R.S. (2007). Mutation of an A-Kinase-Anchoring Protein Causes Long-QT Syndrome. Proc. Natl. Acad. Sci. USA.

[67] Hu Z.-Y., Liu Y.-P., Xie L.-Y., Wang X.-Y., Yang F., Chen S.-Y., Li Z.-G. (2016). AKAP-9 Promotes Colorectal Cancer Development by Regulating Cdc42 Interacting Protein 4. Biochim. Biophys. Acta BBA—Mol. Basis Dis..

[68] Frank B., Wiestler M., Kropp S., Hemminki K., Spurdle A.B., Sutter C., Wappenschmidt B., Chen X., Beesley J., Hopper J.L. (2008). Association of a Common AKAP9 Variant with Breast Cancer Risk: A Collaborative Analysis. JNCI J. Natl. Cancer Inst..

[69] Ciampi R., Knauf J.A., Kerler R., Gandhi M., Zhu Z., Nikiforova M.N., Rabes H.M., Fagin J.A., Nikiforov Y.E. (2005). Oncogenic AKAP9-BRAF Fusion Is a Novel Mechanism of MAPK Pathway Activation in Thyroid Cancer. J. Clin. Investig..

[70] Herter J.M., Grabie N., Cullere X., Azcutia V., Rosetti F., Bennett P., Herter-Sprie G.S., Elyaman W., Luscinskas F.W., Lichtman A.H. (2015). AKAP9 Regulates Activation-Induced Retention of T Lymphocytes at Sites of Inflammation. Nat. Commun..

[71] Uhlen M., Zhang C., Lee S., Sjöstedt E., Fagerberg L., Bidkhori G., Benfeitas R., Arif M., Liu Z., Edfors F. (2017). A Pathology Atlas of the Human Cancer Transcriptome. Science.

[72] Powers A.D., Palecek S.P. (2012). Protein Analytical Assays for Diagnosing, Monitoring, and Choosing Treatment for Cancer Patients. J. Healthc. Eng..

[73] Chandler K., Goldman R. (2013). Glycoprotein Disease Markers and Single Protein-Omics. Mol. Cell. Proteom..

