# Supplementary material for: Changes in Protein Structural Motifs upon Post-Translational Modification in Kidney Cancer
Source: Diagnostics (Basel). 2021 Oct 4;11(10):1836. doi: 10.3390/diagnostics11101836 (PMC8534394; doi:10.3390/diagnostics11101836)
Supplement: Supplementary file 1 [file diagnostics-11-01836-s001.zip › diagnostics-1366565-supplementary.pdf]

Supplementary materials

Table S1. Demography

Case group (kidney cancer)					*Control group (healthy participants)*		
ID	Sex	Years	Stage	TNM	ID	Sex	Years
MMA_001	female	46	IV	T3aN1Mx	MMA_CNT_001	female	53
MMA_002	male	60	I	T1aNxMx	MMA_CNT_002	male	29
MMA_003	female	50	I	T3aN0M0	MMA_CNT_003	female	52
MMA_004	female	57	I	T1aN0Mx	MMA_CNT_004	female	20
MMA_005	female	43	II	T3aN0M0	MMA_CNT_005	male	34
MMA_006	male	68	I	T1bN1Mx	MMA_CNT_006	female	36
MMA_007	male	57	IV	T3aN1M0	MMA_CNT_007	male	36
MMA_008	female	64	I	T1aN0Mx	MMA_CNT_008	male	25
MMA_009	male	48	I	T1bN0M0	MMA_CNT_009	female	48
MMA_010	male	65	I	T2N0M0	MMA_CNT_010	male	30
MMA_011	male	55	I	T1aN0M0	MMA_CNT_011	female	31
MMA_012	male	67	III	T3aN0M0	MMA_CNT_012	female	48
MMA_013	male	61	I	T1aN0Mx	MMA_CNT_013	female	48
MMA_014	male	49	I	T1aN0M0	MMA_CNT_014	female	39
MMA_015	male	60	I	T1aN0M0	MMA_CNT_015	female	26
MMA_016	female	68	I	T1aN0M0	MMA_CNT_016	male	42
MMA_017	male	64	I	T1aN0M0	MMA_CNT_017	male	23
MMA_018	male	63	I	T1aN0Mx	MMA_CNT_018	male	23
MMA_019	female	51	I	T1N0M0	MMA_CNT_019	male	60
MMA_020	male	55	III	T3aN0Mx	MMA_CNT_020	female	34
MMA_021	female	62	I	T3aN0M0	MMA_CNT_021	male	36
MMA_022	female	62	I	T3aN0Mx	MMA_CNT_022	male	26
MMA_023	female	54	I	T3aN0M0	MMA_CNT_023	female	25
MMA_024	male	50	I	T3aN0Mx	MMA_CNT_024	male	31
MMA_025	male	35	I	T3aN0Mx	MMA_CNT_025	male	27
MMA_026	male	55	I	cT3aN0Mx	MMA_CNT_026	male	47
MMA_027	male	73	I	cT1aN0Mx	MMA_CNT_027	male	61
MMA_028	male	72	I	cT1aN0M0	MMA_CNT_028	male	61
MMA_029	male	48	I	T1aN0Mx	MMA_CNT_029	male	62
MMA_030	male	63	I	T3aN0M0	MMA_CNT_030	male	62
MMA_031	female	57	I	T3aN0M0	MMA_CNT_031	male	44
MMA_032	female	60	III	cT3aN0Mx	MMA_CNT_032	male	44
MMA_033	male	56	I	T1aN0M0	MMA_CNT_033	male	51
MMA_034	male	64	IV	T4N1M2	MMA_CNT_034	male	51
MMA_035	male	55	I	T1bN0M0	MMA_CNT_035	male	66
MMA_036	male	41	I	T3aN0Mx	MMA_CNT_036	male	66
MMA_037	male	47	I	T3aN0M0	MMA_CNT_037	male	62
MMA_038	male	35	I	T3aN0Mx	MMA_CNT_038	male	62
MMA_039	male	56	I	T3aNXMx	MMA_CNT_039	male	64
MMA_040	male	64	I	T3aN0M0	MMA_CNT_040	male	56
MMA_041	male	64	III	T3aN0M0			
MMA_042	male	53	I	T1bN0Mx			
MMA_043	male	62	I	T1aN0M0			
MMA_044	male	46	III	T3bN0M0			
MMA_045	male	69	I	T1aN0Mx			

Table 2. List of proteins associated with the development of the kidney cancer. The data was obtained earlier in the study – Arthur Kopylov, Denis Petrovsky, Alexander Stepanov, Vladimir Rudnev, Kristina Malsagova,… and, Anna Kaysheva. Convolutional neural network in proteomics and metabolomics for determination of comorbidity between cancer and schizophrenia, Journal of Biomedical Informatics, Volume 122, 2021, 103890, https://doi.org/10.1016/j.jbi.2021.103890 (https://www.sciencedirect.com/science/article/pii/S1532046421002197).

UniProt, AC	Name	*p*-value (Case vs Control)	Median of Intensity (Case)	Median of Intensity (Control)t	Fold Change
P20851	C4b-binding protein beta chain	0,016951605	2937500	100000	▲29,4
Q08380	Galectin-3-binding protein	0,029654981	2143100	100000	▲21,4
P0C0L5	Complement C4-B	9,98787E-09	628930000	32623000	▲19,3
P80108	Phosphatidylinositol-glycan-specific phospholipase D	0,001844554	1109800	100000	▲11,1
P01019	Angiotensinogen	6,85364E-08	354170000	90310000	▲3,9
P01031	Complement C5	0,012657403	145460000	53810000	▲2,7
P04003	C4b-binding protein alpha chain	0,046634593	185950000	88993000	▲2,1
P01024	Complement C3	1,70093E-10	2625900000	6382300000	▼0,4
P02649	Apolipoprotein E	1,48854E-06	54967000	140130000	▼0,4
P02654	Apolipoprotein C-I	2,34312E-05	42713000	111660000	▼0,4
P02790	Hemopexin	1,37366E-12	1118500000	3094700000	▼0,4
P01011	Alpha-1-antichymotrypsin	3,67231E-10	173040000	485200000	▼0,4
P27169	Serum paraoxonase/arylesterase 1	9,17195E-07	29828000	83893000	▼0,4
P01009	Alpha-1-antitrypsin	1,22346E-08	3061200000	8672400000	▼0,4
P00748	Coagulation factor XII	0,00128007	15873000	45474000	▼0,3
P35542	Serum amyloid A-4 protein	3,3175E-07	7231100	22080000	▼0,3
P02743	Serum amyloid P-component	1,36737E-06	4202600	13082000	▼0,3
O95445	Apolipoprotein M	2,5791E-07	6246100	20132000	▼0,3
P02760	Protein AMBP	6,37869E-09	73793000	241450000	▼0,3
P02768	Albumin	2,70405E-15	65805000000	2,1672E+11	▼0,3
P02647	Apolipoprotein A-I	3,24485E-14	3872500000	12912000000	▼0,3
P19823	Inter-alpha-trypsin inhibitor heavy chain H2	3,67231E-10	188330000	643150000	▼0,3
P10643	Complement component C7	0,001348408	6642700	22747000	▼0,3
P02750	Leucine-rich alpha-2-glycoprotein	3,96051E-08	13150000	45792000	▼0,3
P25311	Zinc-alpha-2-glycoprotein	0,021232926	31893000	114215500	▼0,3
P02746	Complement C1q subcomponent subunit B	0,059918103	2024900	7285200	▼0,3
P06727	Apolipoprotein A-IV	2,32403E-08	112610000	405915000	▼0,3
Q96PD5	N-acetylmuramoyl-L-alanine amidase	2,07973E-07	14459000	52294000	▼0,3
P01860	Immunoglobulin heavy constant gamma 3	5,13769E-14	402300000	1701200000	▼0,2
P08697	Alpha-2-antiplasmin	2,70405E-15	36196000	153170000	▼0,2
Q9NP71	Carbohydrate-responsive element-binding protein	3,18657E-07	7380100	32218000	▼0,2
P02753	Retinol-binding protein 4	2,69585E-09	23930000	111970000	0,2
P06681	Complement C2	1,0718E-06	5627400	26571500	0,2
P02655	Apolipoprotein C-II	9,25956E-09	18718000	96425000	▼0,2
P00734	Prothrombin	2,4742E-12	140880000	747940000	▼0,2

P10909	Clusterin	1,36145E-07	43307000	232340000	▼0,2
P29622	Kallistatin	0,058607463	5157500	33661000	▼0,2
P02763	Alpha-1-acid glycoprotein 1	1,00861E-12	481830000	3262900000	▼0,1
P05090	Apolipoprotein D	1,65511E-09	15495000	115710000	▼0,1
P04196	Histidine-rich glycoprotein	2,41313E-09	35890000	268110000	▼0,1
P00738	Haptoglobin	2,68846E-09	774020000	5860600000	▼0,1
P00736	Complement C1r subcomponent	8,80127E-08	8352300	63958000	▼0,1
P43251	Biotinidase (Biotinase) (EC 3.5.1.12)	0,134029192	100000	799000	▼0,1
P01008	Antithrombin-III	1,65978E-09	47864000	411560000	▼0,1
P19652	Alpha-1-acid glycoprotein 2	5,40809E-15	123110000	1083800000	▼0,1
P02749	Beta-2-glycoprotein 1	4,77346E-09	92660000	845300000	▼0,1
P06396	Gelsolin	2,44106E-09	14839000	145350000	▼0,1
P03952	Plasma kallikrein	1,88806E-08	11157000	124450000	▼0,1
P05155	Plasma protease C1 inhibitor	2,70405E-15	67048000	791170000	▼0,1
P02751	Fibronectin	2,22205E-09	35456000	418550000	▼0,1
Q5T5C0	Syntaxin-binding protein 5	0,522566833	2661100	39434000	▼0,1
P07225	Vitamin K-dependent protein S	1,18951E-07	1035000	19067000	▼0,1
P49908	Selenoprotein P	4,56417E-09	100000	2034400	▼0,0
P08519	Apolipoprotein(a)	1,16081E-06	100000	3207000	▼0,0
Q03591	Complement factor H-related protein 1	4,20862E-07	100000	8945500	▼0,0

Statistical analysis (https://doi.org/10.1016/j.jbi.2021.103890)

Proteins that significantly differentiated studied phenotypes were revealed using a pairwise Student's t-test, and Bonferroni correction for multiple testing was applied to adjust obtained p-values (cut-off $p < 0.05$ and ｜Fold Change｜ > 2). To be selected from the total proteome, the candidate protein should meet the criterion of unicity among the totality identified peptides. Semi-quantitative analysis was conducted in MaxQuant using Intensity and label-free quantitation (LFQ).
